# Molecular Typing of *Burkholderia mallei* Isolates from Equids with Glanders, India

**DOI:** 10.3201/eid2706.203232

**Published:** 2021-06

**Authors:** Harisankar Singha, Mandy C. Elschner, Praveen Malik, Sheetal Saini, Bhupendra N. Tripathi, Katja Mertens-Scholz, Hanka Brangsch, Falk Melzer, Raj K. Singh, Heinrich Neubauer

**Affiliations:** Indian Council of Agricultural Research—National Research Centre on Equines, Haryana, India (H. Singha, S. Saini);; Friedrich-Loeffler-Institut Federal Research Institute for Animal Health, Institute for Bacterial Infections and Zoonoses, Jena, Germany (M.C. Elschner, K. Mertens-Scholz, H. Brangsch, F. Melzer, H. Neubauer);; Ministry of Fisheries, Animal Husbandry & Dairying, Government of India, New Delhi, India (P. Malik);; Indian Council of Agricultural Research, Krishi Bhawan, New Delhi (B.N. Tripathi);; Indian Council of Agricultural Research—Indian Veterinary Research Institute, Izatnagar, India (R.K. Singh)

**Keywords:** glanders, India, *Burkholderia mallei*, bacteria, multilocus sequence typing, multiple-locus variable-number tandem-repeat, MLST, VNTR

## Abstract

We collected 10 *Burkholderia mallei* isolates from equids in 9 districts in India during glanders outbreaks in 2013–2016. Multilocus variable-number tandem-repeat analysis showed 7 outbreak area–related genotypes. The study highlights the utility of this analysis for epidemiologically tracing of specific *B. mallei* isolates during outbreaks.

*Burkholderia mallei* is the etiologic agent of the contagious and fatal infection in equids known as glanders. It is one of the most ancient diseases and is distributed worldwide. *B. mallei* infections are frequently reported in South America, the Middle East, South Asia, and some countries in Africa. Equine glanders is a notifiable zoonotic disease; surveillance measures are enforced by the World Organisation for Animal Health (*1*).

Since 2006, equine glanders has been reported in India with consistently higher numbers from the Uttar Pradesh state (*2*,*3*). Regular glanders surveillance programs revealed presence of the disease in 14 states and, during 2015–2018, fresh *B. mallei* infections were reported in 6 states: Jammu and Kashmir, Gujarat, Rajasthan, Delhi, Madhya Pradesh, and Tamil Nadu (*4*). Epidemiologic investigations indicated that trading of equids from Uttar Pradesh to other states played a major role in spreading glanders (*2*). However, *B. mallei* isolates were not genotyped, which is necessary for understanding the epidemiologic association between glanders outbreaks across India.

Our study describes molecular typing of 10 *B. mallei* isolates recovered from horses (n = 4) and mules (n = 6) during 2013–2016 (Table; Appendix 1 Figure). All the affected equids were used for cart pulling and kept in small household stables. Five isolates (3324, 3478, 3701, 3711, and 3712), originating from 3 horses and 2 mules, were from adjoining districts of Uttar Pradesh state, which is regarded as a glanders hotspot zone (*2*). Three isolates (3076, 3081, 3595) from mules were located in 2 districts of Himachal Pradesh. Available information from the equine keeper suggested that these animals were traded from Uttar Pradesh and were responsible for the reported glanders incidence in this state. One isolate was recovered from a mule (3880) in Gujarat and 1 from a horse (3897) in Haryana state; both animals had no recent travel history.

**Table:** Location, host, and isolation year of 10 *Burkholderia mallei* isolates included for molecular typing, India

*B. mallei* isolate	Place of origin (district, state)	Year isolated	Host species	Salient clinical signs	Sample type
India3076	Solan, Himachal Pradesh	2013	Mule	Blood tinged nasal discharge, respiratory distress	Nasal swab
India3081	Solan,Himachal Pradesh	2013	Mule	Respiratory distress, nasal discharge, cutaneous nodules	Nasal swab
India3324	Hardoi,Uttar Pradesh	2014	Horse	Nasal discharge, hind limb ulcer, liver abscess	Liver abscess
India3478	Agra, Uttar Pradesh	2014	Horse	Hind limb ulceration, lacrimation	Lesion swab
India3595	Mandi, Himachal Pradesh	2015	Mule	Labored breathing, nasal discharge	Nasal swab
India3701	Kasganj,Uttar Pradesh	2015	Mule	Nasal discharge, cutaneous nodules	Lesion swab
India3711	Etah, Uttar Pradesh	2015	Mule	Respiratory distress, cutaneous nodules	Nasal swab
India3712	Ghaziabad, Uttar Pradesh	2015	Horse	Ulcerous nodules on body surface	Nasal swab
India3880	Banaskantha, Gujarat	2016	Mule	Mucopurulent nasal discharge	Nasal swab
India3897	Yamunanagar, Haryana	2016	Horse	Ulcerous nodules on hind limb and forelimb, purulent nasal discharge	Lesion swab

The isolates were recovered from different types of biologic samples (Table) as described previously (*3*) and identified as *B. mallei* by real-time PCR (*1*). Genomic DNA was extracted by using the PureLink genomic DNA isolation kit (Invitrogen, https://www.thermofisher.com) and used for PCR-based multilocus sequence typing (MLST) and multilocus variable-number tandem-repeat (VNTR) analysis (MLVA). We typed all 10 *B. mallei* isolates as sequence type (ST) 40 by the *B. pseudomallei* MLST scheme and ST734 by the *B. cepacia* MLST scheme (*5*,*6*); Appendix 2 Tables 1, 2).

We conducted MLVA by PCR amplification and sequencing of 23 loci using previously described primers (*7*). We determined sequence length and repeat number for each locus using Geneious software version 6.1.8 (https://www.geneious.com). A distance matrix giving the number of VNTR loci differing between isolates was used for analysis applying the minimum-evolution method implemented in MEGA X software version 10.0.5 (https://www.megasoftware.net).

MLVA assigned the 10 isolates to 7 genotypes, indicating considerable variability among *B. mallei* isolates in India (Figure, panel A). Identical MLVA patterns were observed for isolates 3076 and 3081 from Himachal Pradesh and isolate 3324 from Uttar Pradesh. These findings correlate with epidemiologic investigations regarding the spread of a particular strain of *B. mallei* by equine movement, emphasizing the need to control equine trade between states. An identical pattern was also observed for *B. mallei* 3701 and 3712, which were isolated from Kasganj and Ghaziabad districts, 190 km apart in Uttar Pradesh state.

**Figure:**
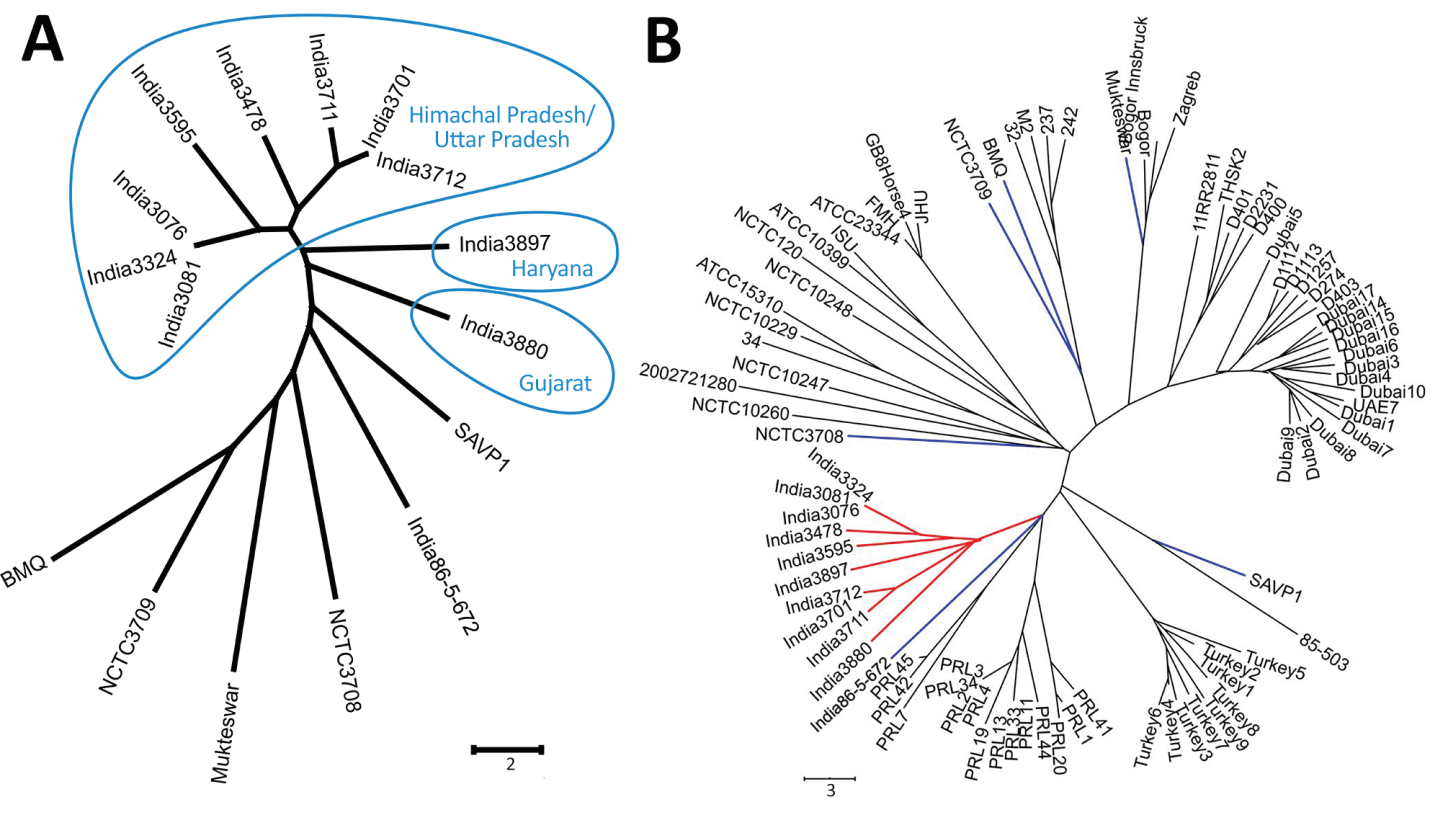
Minimum evolution trees based on 23 VNTR loci of 10 *Burkholderia mallei* isolates from Himachal Pradesh, Uttar Pradesh, Gujarat, and Haryana states, India, compared with reference sequences. A) Comparison of Himachal Pradesh–Uttar Pradesh cluster isolates (blue circles) with 6 older *B. mallei* isolates from India. B) Comparison of Himachal Pradesh–Uttar Pradesh cluster isolates (red branches) with 77 previously published *B. mallei* isolates, including the 6 others from India (blue branches). Scale bars indicate allelic differences.

The isolates 3897 and 3880 from Haryana and Gujarat differ clearly from the isolates from Himachal Pradesh–Uttar Pradesh cluster (Figure, panel A). However, isolates 3712, 3880, and 3897 were previously grouped into the L2B2sB2 branch by HRM-PCR analysis (*8*), which indicates superiority of MLVA for better epidemiologic resolution of glanders outbreaks.

Comparative MLVA between old and recent isolates from India revealed that most of the earlier isolates Mukteswar, BMQ, NCTC3708, NCTC3709, and India 86–567–2 are distantly related, whereas the isolate SAVP1 showed the highest similarity to the new isolates (Figure, panel A; Appendix 2 Table 3).

Further analysis of these *B. mallei* isolates plus 77 from other countries revealed that the 10 recent isolates of our study form a cluster that is most similar to isolates from Pakistan, followed by isolates from Turkey (Figure, panel B). This finding suggests that *B. mallei* strains prevalent in geographically close countries might have originated from an ancestral clone and gradually disseminated to different areas. Of interest, adoption of a strict regulatory movement policy at the beginning of the 19th century for control and eradication of glanders might have resulted in establishing specific *B. mallei* lineages at different ecologic settings. Our finding confirms previous observations regarding circulation of different *B. mallei* MLVA types in the Middle East (*9*,*10*).

In summary, MLVA proved useful as a genetic tool for classifying of *B. mallei* isolates and tracing possible infection chains of glanders outbreaks in equids. VNTR information from more *B. mallei* isolates from India and other countries would be helpful to draw an epidemiologic conclusion between outbreaks.

Appendix 1 Additional information about distribution of *Burkholderia mallei* isolates obtained from equids with glanders, India.

Appendix 2 Additional information about molecular typing of *Burkholderia mallei* isolates obtained from equids with glanders, India.
