# Supplementary material for: Molecular Typing of Burkholderia mallei Isolates from Equids with Glanders, India
Source: Emerg Infect Dis. 2021 Jun;27(6):1745–8. doi: 10.3201/eid2706.203232 (PMC8153868; doi:10.3201/eid2706.203232)
Supplement: Appendix 1 — Additional information about distribution of Burkholderia mallei isolates obtained from equids with glanders, India. [file 20-3232-Techapp-s1.pdf]

Article DOI: https://doi.org/10.3201/eid2706.203232

Molecular Typing of *Burkholderia mallei* Isolates from Equids with Glanders, India

Appendix 1

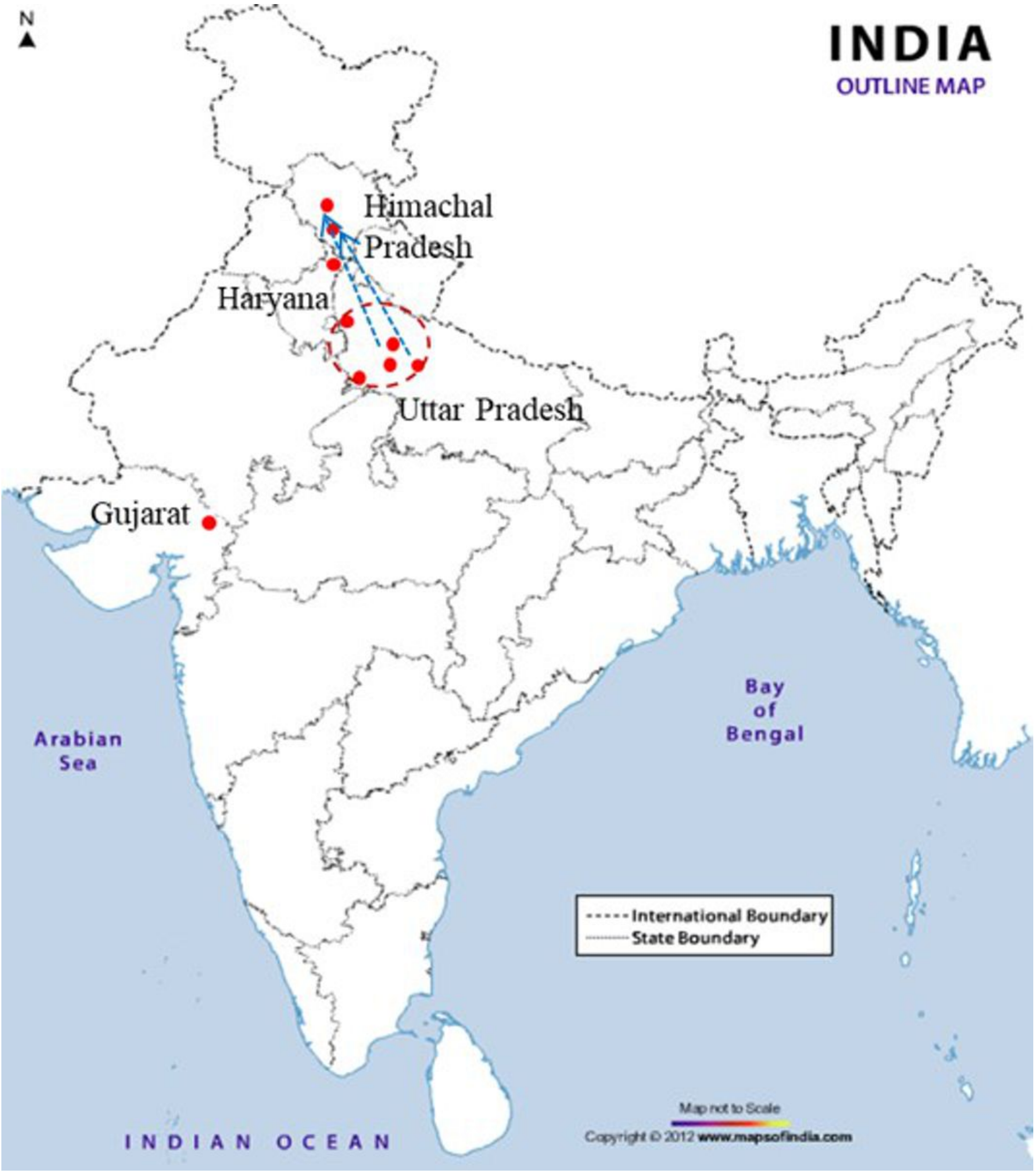

Appendix 1 Figure. Distribution of the *Burkholderia mallei* isolates in Himachal Pradesh, Uttar Pradesh, Gujarat, and Haryana states included in this study. The dashed circle indicates glanders hotspot zone encompassing 14 districts (~55,000 km^2) in western part of Uttar Pradesh. Dashed arrow suggests interstate migration of equids implicated in glanders outbreak in Himachal Pradesh. Source: https://www.mapsofindia.com.
